# Severe Covid-19 in pregnant and postpartum women admitted to an intensive care unit: A retrospective cohort study

**DOI:** 10.1371/journal.pone.0295444

**Published:** 2023-12-14

**Authors:** Isabel Cristina Melo Mendes, Ana Luiza Martins de Oliveira, Priscila Martins Pinheiro Trindade, Wallace Mendes da Silva, Patricia Pontes Frankel, Carolina Carvalho Mocarzel, Marta de Alencar Rosa, Ana Paula Santos Nascimento, Glaucia de Melo Rodrigues, Clarisse Pimentel, Raissa de Moraes Perlingeiro, Alessandra Gonçalves Lisbôa Pereira, Claudia Caminha Escosteguy, Rafael Mello Galliez

**Affiliations:** 1 Infectious Diseases Post-Graduation Program, Federal University of Rio de Janeiro, Rio de Janeiro, Brazil; 2 Instituto Estadual de Infectologia São Sebastião, Rio de Janeiro, Brazil; 3 Faculty of Pharmacy, Fluminense Federal University, Niteroi, Brazil; 4 Hospital Federal dos Servidores do Estado, Rio de Janeiro, Brazil; 5 Faculty of Medicine, Estácio de Sá University (UNESA/IDOMED), Rio de Janeiro, Brazil; 6 Faculty of Medicine, Federal University of Rio de Janeiro, Rio de Janeiro, Brazil; 7 Center for Response and Studies on Emerging and Reemerging Infectious Diseases (NEEDIER), Federal University of Rio de Janeiro, Rio de Janeiro, Brazil; Universidade Federal do Espirito Santo, BRAZIL

## Abstract

**Background:**

SARS-CoV-2 infection is associated with worse maternal and fetal outcomes. This study aims to describe the characteristics of pregnant and postpartum women with severe Covid-19 admitted to ICU.

**Methods and findings:**

It’s a retrospective cohort study evaluating pregnant and postpartum women referenced to a specialized ICU between May 2020 and June 2022. Covid-19 was confirmed with RT-PCR or rapid antigen test on a nasopharyngeal swab. Variables were described by median and IQR when numerical, and by frequency and percentage when categorical. OR with 95% CI were calculated for the evaluation of factors related to death. P-values were calculated using Pearson’s ꭓ^2^-test, Fisher’s exact test, Wilcoxon rank sum test, and Kruskall-Wallis test, and statistical significance was established as < 0·05. Missing data were excluded. All statistical analysis were performed using R software version 4.2.2. Of the 101 admissions, 85 (84·2%) were of pregnant women. Obesity (23·0%) and systemic arterial hypertension (13·0%) were the most prevalent medical conditions. Sixty-six (65·3%) were admitted using some type of oxygen support. Forty-seven (46·5%) evolved to mechanical ventilation. There were 61 events considered obstetric complications, with 8 stillbirths/fetal losses. The overall lethality was 15·8%. Pregnancy interruption, need for non-invasive mechanical ventilation, level of oxygen support at admission, prone maneuver, hemodialysis, and healthcare-related infections were factors associated with death. Evaluating the WHO 7-category ordinary scale, there was a trend of increase in the risk of death with higher punctuation, with a statistically significant difference of women with 5 (OR = 7·27; 95% IC = 1·17–194; p = 0·031) or 6 points (OR = 12·0; 95% IC = 1·15–391; p = 0·038) when compared to the ones with 3 points, i.e., of women admitted with a high-flow non-rebreathing mask or invasive mechanical ventilation, compared with the ones admitted at room air, respectively. The main limitations of this study are the relatively small number of participants, and the use of data derived of medical records—which are susceptible to misclassification and variable amounts of missing data.

**Conclusions:**

Pregnant and postpartum women with severe Covid-19 have high lethality and a high incidence of clinical and obstetric complications. These findings support that this population should be prioritized in public health strategies that address Covid-19.

## Introduction

Among the groups considered as having a higher risk of severe Covid-19, pregnant and postpartum women are considered especially vulnerable. Initial reports suggested that there wasn’t an association of pregnancy and severe Covid-19 [1]. At the same time, they are frequently excluded from clinical trials, resulting in a lack of evidence about the safety and efficacy of treatments in this population. These facts have led to a delay in the incorporation of therapeutic and/or preventive strategies for this group and in the development of specific public health policies [2, 3].

New data have shown that pregnant women with Covid-19 have a bigger risk of hospitalization, intensive care unit (ICU) admission, and need for invasive mechanical ventilation (IMV) when compared with non-pregnant women of childbearing age [4–6]. There is also growing evidence of a higher incidence of obstetric complications, and unfavorable maternal outcomes in women with Covid-19 during pregnancy [7–10]. Several risk factors to moderate and severe Covid-19 in pregnancy have been demonstrated in different studies, like age, being a healthcare worker, presence of comorbidities, gestational age, race, and low socioeconomic status [6, 11–13].

In Brazil, maternal mortality due to Covid-19 is considered elevated. At the end of May 2020, the Brazilian Ministry of Health has reported the occurrence of 36 maternal deaths due to Covid-19, that have occurred up to the epidemiological week 21 of that year. At the time, this number placed Brazil as the leader in maternal deaths due to the disease in the world [14]. Posterior data have shown 549 Covid-19 deaths of pregnant and postpartum women in 2020, with an average of 12.1 deaths per week. This represented a fatality rate of 7.2%, while the fatality rate of Covid-19 for the general population in the country was of 2.6% [15].

Investigations regarding factors associated with maternal and postpartum death related to Covid-19 have shown being a black woman, living in a rural area and being hospitalized outside the residence municipality as risk factors for death in this population. Odds of hospitalization, ICU admission, and use of invasive ventilatory support were found to be higher among maternal deaths than in the control group [15]. Maternal mortality can also be influenced by the quality of care, including adequate and timely access, and availability of necessary resources. In this context, interruption of regular antenatal care caused by the pandemic and the lack of ICU beds for obstetric patients may have contributed to excessive death among pregnant and postpartum women in Brazil, leading to important delays in receiving appropriate care at the right time [14, 15].

The State Institute of Infectious Diseases São Sebastião (Instituto Estadual de Infectologia São Sebastião—IEISS) is a reference center for infectious diseases in the city of Rio de Janeiro, Brazil. With 16 ICU beds, it was part of the state of Rio de Janeiro (RJ) response to the Covid-19 pandemic, receiving pregnant and postpartum women from different regions of the state.

Although the body of evidence about clinical characteristics and the course of the disease has grown exponentially in the last few years, information regarding severe Covid-19 in pregnancy is still scarce. The primary objective of this study is to analyze lethality related to Covid-19 in pregnant and postpartum women. The secondary objectives are to analyze clinical, laboratory, and sociodemographic characteristics, and possible prognostic factors associated with death in this population.

## Methods

### Study’s design, location, and population

It’s a retrospective exploratory cohort study evaluating pregnant and postpartum women admitted to the ICU of IEISS due to confirmed or suspected Covid-19 between May 2020 and June 2022.

A case was determined as microbiological confirmed if the patient presented a positive diagnostic test, either RT-PCR or rapid antigen test (Panbio^™^, Abbott, IL, USA; sensitivity = 91.4% and specificity = 99.8%) [16] on nasopharyngeal swabs. Since many women were admitted late in the disease, cases with negative results had their notification and medical records reviewed for evaluation of previous positive results from the health unit that requested transference to the ICU and for the clinical possibility of diagnosis. Cases were classified as non-microbiological confirmed if there were clinical, epidemiological, and radiological characteristics compatible with Covid-19, but lacked a positive test in our or in the primary health unit. Cases that had no history of a positive test and that had other etiology as more probable were excluded.

Consecutive admissions in the ICU were evaluated. Follow-up time was from admission to discharge of the ICU or death.

### Data collection and variables of interest

The collected data were composed of clinical, laboratory, radiological and sociodemographic information available from the medical and multidisciplinary records of each patient. Procedures used to classify cases were described above. Data were collected by health professionals of the institution, familiar with medical terms, after standardization for collection and recording of the data.

The following variables were considered of interest:

- Sociodemographic: age, race, and distance from patient’s residence and health unit of origin to the IEISS;
- Clinical: the presence of comorbidities, symptoms duration until admission to the ICU, immunization status at admission, need for oxygen support, laboratory evidence of Covid-19, the requirement of non-invasive (NIMV) and invasive mechanical ventilation (IMV), time in IMV, time to start IMV since admission, prone maneuver, the realization of tracheostomy, the occurrence of thromboembolic events, the requirement of hemodialysis, ketoacidosis at admission, the occurrence of healthcare-related infections, length of hospitalization and final outcome (discharge vs. death);
- Obstetric: pregnancy status at admission (pregnant vs. postpartum), gestational age at admission, pregnancy interruption, time until interruption, mode of delivery, obstetric complications, and stillbirth/fetal loss during hospitalization.

Race registers were self-reported or defined by the attending physician. Distances were estimated in kilometres using the freely available Google Maps^®^ app. The patients were considered immunized if, at admission, they had all recommended doses according to the type of vaccine. Partially or non-vaccinated women were considered as non-immunized. For confirmation of vaccination status, records of the National Immunization Program (Programa Nacional de Imunização—PNI), a national electronic based-system with records of all Covid-19 vaccinations of the country, were consulted.

Occurrence of ketoacidosis was defined as the concomitant presence of metabolic acidosis (serum bicarbonate < 22 mEq/L) and an elevated anion gap (> 10 mEq/L), with or without ketonuria on a urine test collected within 72h from admission. Preterm births were defined as births with less than 37 weeks of pregnancy, irrespective of the mode of delivery. Stillbirths and fetal losses were counted together.

The periods of predominance of SARS-CoV-2 variants were defined according to the genomic surveillance data from the state of Rio de Janeiro. One lineage was classified as predominant when its detection rate was bigger than 90% on individuals in a period. According to those data, the original lineage was circulating in Rio de Janeiro between March and December 2020, P2 was circulating between December 2020 and February 2021, Gamma was circulating between March and June 2021, Delta was the predominant variant between August and November 2021, and Omicron was the main variant between January and March 2022 [17]. Information about the proportion of the population of the city of Rio de Janeiro vaccinated was obtained through the official epidemiological reports [18].

The authors responsible for the collection of data had access to information that could identify individual participants during or after data collection. Data were accessed from 1^st^ July 2022 to 30^th^ November 2022.

### Statistical analysis

For descriptive analysis, numerical variables were described by median and IQR, while categorical variables were described by frequency and percentage. Odds ratios (OR) with 95% confidence intervals (IC 95%) were calculated for the evaluation of factors related to death. P-values were calculated using Pearson’s ꭓ^2^-test, Fisher’s exact test, Wilcoxon rank sum test, and Kruskall-Wallis test, and statistical significance was established as < 0·05. Missing data were excluded from the analysis. Multivariable analysis was not performed. The datasets with the variables collected were built using the Google Sheet^®^ and Excel^®^ applications. The data were anonymized and exported to R software as.cvs files. All statistical analyses were performed using R software version 4.2.2 (R Foundation for Statistical Computing).

### Ethical aspects

This study was approved by the Research Ethics Committee of the Hospital Federal dos Servidores do Estado according to all Ethical in Human Research regulations (numbers 30161620.0.0000.5257, 30127020.0.0000.0068 and 48749021.3.0000.5252). A consent form for the participants was not required due to the secondary origin of the data. The present study followed the recommendations presented in the “Strengthening of the Reporting of Observational Studies in Epidemiology” (STROBE) statement.

## Results

### General and baseline characteristics

Overall, there were 104 admissions during the established timeframe, corresponding to data from 103 different women. After reviewing the notification sheets and medical records, 3 women were excluded from the analysis since they were considered as having another cause for admission. The characteristics of the 101 admissions evaluated are summarized in Table 1.

**Table 1:** Patients’ characteristics.

Variables	Overall, N = 101^1^	Discharge, N = 85^1^	Death, N = 16^1^	p-value^2^
**Age**	30.0 (25.0, 34.0)	30.0 (25.0, 34.0)	30.0 (26.5, 33.5)	> 0.9
**Race**				0.9
White	43/88 (49%)	36/73 (49%)	7/15 (47%)	
Non-white	45/88 (51%)	37/73 (51%)	8/15 (53%)	
**Number of comorbidities**				0.3
0	53/100 (53%)	48/85 (56%)	5/15 (33%)	
1	33/100 (33%)	26/85 (31%)	7/15 (47%)	
2	8/100 (8.0%)	6/85 (7.1%)	2/15 (13%)	
At least 3	6/100 (6.0%)	5/85 (5.9%)	1/15 (6.7%)	
**Distance from home to the ICU (Km)**	37.0 (24.0, 65.0)	38.0 (24.0, 67,0)	24.0 (18.0, 41.0)	**0.047**
**Pregnancy status**				0.13
Postpartum	16/101 (16%)	11/85 (13%)	5/16 (31%)	
Pregnant	85/101 (84%)	74/85 (87%)	11/16 (69%)	
**Gestational age at admission**	29.0 (24.0, 34.0)	29.0 (24.0, 33.0)	29.0 (24.0, 34.0)	0.5
**Duration of symptoms (days)**	9.0 (6.0, 11.0)	9.0 (6.0, 11.8)	8.5 (7.0, 10.0)	0.9
**Pregnancy interruption**	48/101 (48%)	34/85 (40%)	14/16 (88%)	**< 0.001**
**Gestational age at interruption**	34.0 (31.0, 35.0)	33.5 (31.0, 35.0)	34.5 (33.2, 35.0)	0.4
**Mode of delivery**				0.7
Cesarian	41/48 (85%)	28/34 (82%)	13/14 (93%)	
Normal	7/48 (15%)	6/34 (18%)	1/14 (7.1%)	
**7-category scale (WHO)**				0.051
3	25/91 (27%)	24/75 (32%)	1/16 (6.2%)	
4	27/91 (30%)	23/75 (31%)	4/16 (25%)	
5	31/91 (34%)	23/75 (31%)	8/16 (50%)	
6	8/91 (8.8%)	5/75 (6.7%)	3/16 (19%)	
**Non-invasive mechanical ventilation (NIMV)**	50/97 (52%)	35/81 (43%)	15/16 (94%)	**< 0.001**
**Invasive mechanical ventilation (IMV)**	47/101 (47%)	31/85 (36%)	16/16 (100%)	**< 0.001**
**Time on IMV (days)**	12.0 (6.0, 23.0)	13.0 (6.5, 26.5)	12.0 (5.5, 16.8)	0.5
**Prone manouver**	20/45 (44%)	10/30 (33%)	10/15 (67%)	0.056
**Tracheostomy**	11/46 (24%)	8/30 (27%)	3/16 (19%)	0.7
**Thrombotic event**	6/99 (6.1%)	4/84 (4.8%)	2/15 (13%)	0.2
**Presence of ketonuria**	19/54 (35%)	17/46 (37%)	2/8 (25%)	0.7
**Presence of metabolic acidosis**	72/94 (77%)	60/80 (75%)	12/14 (86%)	0.5
**Hemodialysis**	13/99 (13%)	7/83 (8.4%)	6/16 (38%)	**0.006**
**Obstetric complications**	50/92 (54%)	37/78 (47%)	13/14 (93%)	**0.002**
**Healthcare-related infections**	29/100 (29%)	19/85 (22%)	10/15 (67%)	**0.001**
**Length of stay (days)**	8.0 (4.0, 21.0)	7.0 (4.0, 17.0)	16.0 (10.8, 22.0)	**0.004**
**Immunization status**				0.6
Non-immunized	90/97 (93%)	74/81 (91%)	16/16 (100%)	
Immunized	7/97 (7.2%)	7/81 (8.6%)	0/16 (0%)	

^1^Median (p25, p75) or n/N (%)

^2^Wilcoxon rank sum test; Pearson’s Chi-squared test; Fisher’s exact test

Of the 101 admissions, 85 were of pregnant women at admission, and 16 were of postpartum women that had their delivery in another health unit. The median age was 30 years old (IQR = 25–34). From the 88 women with information registered regarding ethnicity, 43 were white, and 45 were non-white. The majority of the women evaluated did not have any comorbidities. Among the ones with a known health problem, obesity and systemic arterial hypertension were the most prevalent medical conditions (Table 2).

**Table 2:** Description of comorbidities.

Comorbidity	n/N (%)
**Obesity**	23/100 (23%)
**Sistemic arterial hypertension**	13/100 (13%)
**Asthma**	7/100 (7%)
**Diabetes**	4/100 (4%)
**Bronchitis**	3/100 (3%)
**Sickle cell disease**	2/100 (2%)
**Syphilis**	2/100 (2%)
**Others**	13/100 (13%)
**None**	53/100 (53%)

The median time from the beginning of symptoms until admission to the ICU was of 9 days (IQR = 6–11), indicating that most were on the second week of the disease. Regarding ventilatory status, 66 were admitted using some type of oxygen support, although only 8 were admitted already on mechanical ventilation. The median time of hospitalization was 8 days (IQR = 4–21).

### Immunization status

Of the 97 women with information about Covid-19 vaccination, the majority were non-immunized, with only 7 being fully immunized at admission. Even when excluding the 17 admissions that occurred before February 2021, when Covid-19 vaccination was made available for the general population in Rio de Janeiro, only a small percentage of the women could be considered fully immunized (7/82; 8·53%). Of the remaining women admitted in this period, 68 (82·92%) didn’t have any dose of any type of immunizer against Covid-19, and 7 (8·53%) were only partially immunized, having not completed the recommended vaccine schedule.

### Clinical and obstetric complications

At the first days of hospitalization, 72 patients had metabolic acidosis, with 61 presenting an elevated anion gap, and 14 presenting both characteristics and ketonuria. During the period of the study, 6 thrombotic events were identified or suspected, and 13 patients were placed on hemodialysis. Twenty-nine patients had at least one event of healthcare-related infection.

Forty-seven women evolved to mechanical ventilation (35 pregnant and 12 postpartum), with a median time from admission to IMV of 2 days (IQR = 1–3) and a median time of 12 days (IQR = 6–23) on this modality of respiratory support. Of these 47 patients, 11 were submitted to tracheostomy and 20 were positioned in prone decubitus at least once.

Of the 85 pregnancies, 33 interruptions were identified during hospitalization. Of the women that were admitted at the postpartum period, 15 had their pregnancies interrupted in the primary health unit. In both groups, cesarian was the main mode of delivery. Of these 48 women with a history of interruption, 38 were intubated at some point during hospitalization (26 were admitted while pregnant and 12 in puerperium).

There were 61 events considered obstetric complications, mainly preterm birth, with hypertensive disorders of pregnancy being the second most frequent. There were 8 stillbirths/fetal losses that occurred during hospitalization of women who were pregnant at admission (Table 3).

**Table 3:** Description of obstetric complications.

Obstetric complications	n/N (%)
**Preterm birth**	36/92 (39,1%)
**Hypertensive disorders of pregnancy**	9/92 (9,8%)
**Stillbirth/fetal loss**	8/92 (8,7%)
**Placental abruption**	4/92 (4,3%)
**Spontaneous abortion**	1/92 (1,1%)
**Corioamnionitis**	1/92 (1,1%)
**Oligohydramnios**	1/92 (1,1%)
**Acute fatty liver of pregnancy**	1/92 (1,1%)

### Clinical outcomes

Overall, there were 16 deaths, 5 in the group of postpartum women and 11 in the group that was admitted pregnant, which corresponds to an overall lethality of 15·8%. Comparing pregnancy and postpartum status, lethality was higher in the last group (12·9 [11/85] vs. 31·2% [5/16], respectively). However, the lethality of the women that needed interruption of pregnancy during hospitalization at the ICU was 29·2% (14/48), lower than the one observed in the postpartum group. Among the women that needed mechanical ventilation, lethality was 34·0%.

Comparing the patients that were discharged with the ones that have died, both groups were similar in most characteristics. The group with unfavorable outcomes had a greater proportion of pregnancy interruption, a higher frequency of NIMV, IMV, prone, hemodialysis, and healthcare-related infections, and a longer length of hospitalization. The group of women that survived had a bigger proportion of the further distance from their municipality to the ICU. There was no statistically significant difference between groups regarding other variables, such as gestational age at admission, age, time from the beginning of symptoms and admission, and time on IMV.

All women that died needed mechanical ventilation. Pregnancy interruption (OR = 9·70; 95% IC = 2·46–70·4; p < 0·001), receiving NIMV (OR = 17·1; 95% IC = 3·20–427; p < 0·001), being submitted to prone maneuver (OR = 3·82; 95% IC = 1·04–15·7; p = 0·043), receiving hemodialysis (OR = 6·33; 95% IC = 1·69–23·7; p = 0·007), and having at least one episode of healthcare-related infection (OR = 6·69; 95% IC = 2·08–24·4; p = 0·001) were factors statistically associated with death (Table 4).

**Table 4:** Variated analysis of participants characteristics.

	Discharge	Death	OR	p.ratio
	N = 85	N = 16		
**Age**	30.0 (25.0, 34.0)	30.0 (26.5, 33.5)	1.00 (0.92, 1.09)	0.989
**Race**				
White	36 (49.3%)	7 (46.7%)	Ref.	Ref.
Non-white	37 (50.7%)	8 (53.3%)	1.11 (0.35, 3.54)	0.859
**Number of comorbidities**				
0	48 (56.5%)	5 (33.3%)	Ref.	Ref.
1	26 (30.6%)	7 (46.7%)	2.53 (0.72, 9.62)	0.147
2	6 (7.06%)	2 (13.3%)	3.21 (0.35, 20.1)	0.267
At least 3	5 (5.88%)	1 (6.67%)	2.05 (0.07, 17.8)	0.599
**Distance from home to ICU (Km)**	38.0 (24.0, 67.0)	24.0 (18.0, 41.0)	0.99 (0.97, 1.01)	0.225
**Pregnancy status**				
Postpartum	11 (12.9%)	5 (31.2%)	Ref.	Ref.
Pregnant	74 (87.1%)	11 (68.8%)	0.33 (0.10, 1.24)	0.096
**Gestational age at admission**	29.0 (24.0, 33.0)	29.0 (25.0, 34.0)	1.04 (0.94, 1.15)	0.480
**Duration of symptoms (days)**	9.00 (6.00, 11.8)	8.50 (7.00, 10.0)	0.99 (0.86, 1.14)	0.846
**Pregnancy interruption**	34 (40.0%)	14 (87.5%)	9.70 (2.46, 70.4)	**< 0.001**
**Gestational age at interruption**	33.5 (31.0, 35.0)	34.5 (33.2, 35.0)	1.13 (0.88, 1.46)	0.327
**Mode of delivery**				
Cesarian	28 (82.4%)	13 (92.9%)	Ref.	Ref.
Normal	6 (17.6%)	1 (7.14%)	0.40 (0.01, 2.82)	0.402
**7-category scale (WHO)**				
3	24 (32.0%)	1 (6.25%)	Ref.	Ref.
4	23 (30.7%)	4 (25.0%)	3.72 (0.47, 107)	0.231
5	23 (30.7%)	8 (50.0%)	7.27 (1.17, 194)	**0.031**
6	5 (6.67%)	3 (18.8%)	12.0 (1.15, 391)	**0.038**
**Non-invasive mechanical ventilation (NIMV)**	35 (43.2%)	15 (93.8%)	17.1 (3.20, 427)	**< 0.001**
**Invasive mechanical ventilation (IMV)**	31 (36.5%)	16 (100%)	.	.
**Time on IMV (days)**	13.0 (6.50, 26.5)	12.0 (5.50, 16.8)	0.98 (0.94, 1.03)	0.475
**Prone manouver**	10 (33.3%)	10 (66.7%)	3.82 (1.04, 15.7)	**0.043**
**Tracheostomy**	8 (26.7%)	3 (18.8%)	0.66 (0.12, 2.82)	0.583
**Thrombotic event**	4 (4.76%)	2 (13.3%)	3.12 (0.36, 18.9)	0.267
**Presence of ketonuria**	17 (37.0%)	2 (25.0%)	0.60 (0.07, 3.06)	0.558
**Presence of metabolic acidosis**	60 (75.0%)	12 (85.7%)	1.88 (0.45, 14.1)	0.417
**Hemodialysis**	7 (8.43%)	6 (37.5%)	6.33 (1.69, 23.7)	**0.007**
**Obstetric complications**	37 (47.4%)	13 (92.9%)	12.5 (2.30, 316)	**0.001**
**Healthcare-related infections**	19 (22.4%)	10 (66.7%)	6.69 (2.08, 24.4)	**0.001**
**Length of stay (days)**	7.00 (4.00, 17.0)	16.0 (10.8, 22.0)	1.03 (1.00, 1.07)	**0.042**
**Immunization status**				
Non-immunized	74 (91.4%)	16 (100%)	Ref.	Ref.
Immunized	7 (8.64%)	0 (0.00%)	.	.

Evaluating the WHO 7-category ordinary scale, there was a trend of increase in the risk of death with higher punctuation, with a statistically significant difference of women with 5 (OR = 7·27; 95% IC = 1·17–194; p = 0·031) or 6 points (OR = 12·0; 95% IC = 1·15–391; p = 0·038) when compared to the ones with 3 points, i.e., of women admitted with a high-flow non-rebreathing mask or invasive mechanical ventilation, compared with the ones admitted at room air, respectively.

### Trends across time and according to SARS-CoV-2 variants

**Fig 1:**
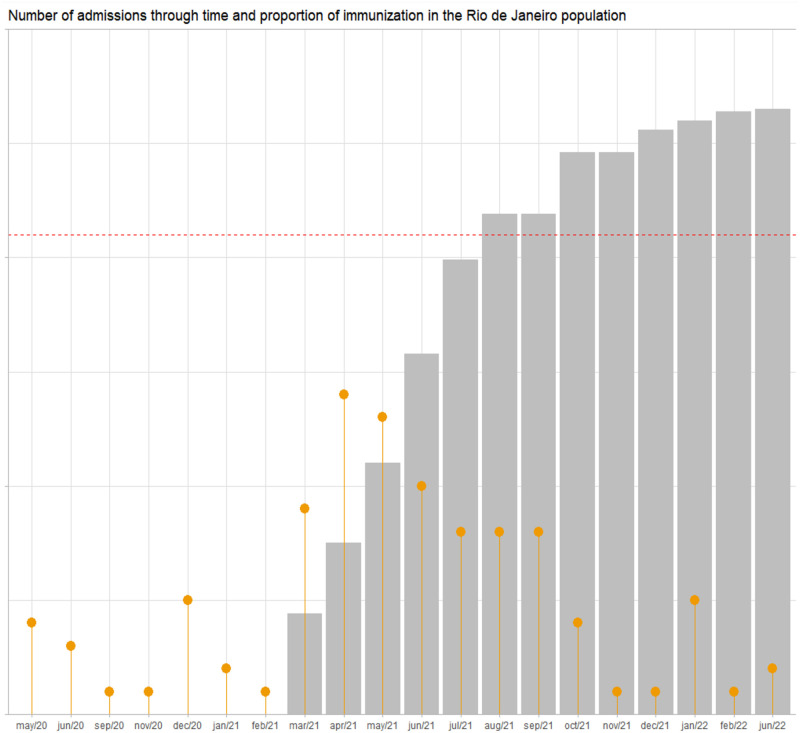
Number of admissions (lollipop graph) and proportion of the vaccinated population in the city of Rio de Janeiro (bar graph) through time. The red dotted line represents a 70% threshold of the general population vaccinated.

Most of the admissions occurred in 2021, especially in the period from March to September, with the highest number of admissions in the months of April and May 2021. According to surveillance data, this period corresponds to the ones when Gamma and, posteriorly, Delta were the predominant variants circulating in the city. There was a trend to stabilization and then a decrease in admissions, following the increase in the proportion of the population that was immunized against Covid-19 (Fig 1).

When analyzing the distribution of fatal cases according to variants’ circulation, the period with the highest lethality was the one when Delta was the predominant variant, with 5 deaths among the 21 admissions in the period (23·8%), followed by the period when the Gamma variant was the main one (5/48; 10·4%). There were no deaths during the P2 and Omicron waves. There were no deaths registered from October 2021 onwards.

## Discussion

Our results show that severe Covid-19 in women that are pregnant or in the postpartum period is associated with high lethality (15·8%). Lethality was even higher among the ones that required IMV (34·0%). A greater proportion of pregnancy interruption, a higher frequency of NIMV, IMV, prone, hemodialysis, healthcare-related infections, and a longer length of hospitalization were found in the group of patients that have died. Pregnancy interruption, receiving NIMV, being submitted to prone maneuver, receiving hemodialysis, and having at least one episode of healthcare-related infection were factors that were found to be statistically associated with death.

Previous studies have shown a higher risk of severe disease, hospital and ICU admissions, and death in this population [4–6, 19, 20]. Maternal mortality due to Covid-19 in Brazil is alarmingly high, although specific data regarding these patients are still scarce. According to a report by the Pan-American Health Organization (OPAS) in 2021, maternal lethality in Brazil due to Covid-19 was 7·2%, while global lethality in the country due to SARS-CoV-2 infection was 2·8% in the same period [21].

The Obstetric Observatory Brazil—COVID-19 (OOBr COVID-19) is part of the Brazilian Obstetric Observatory, a project that has as its objective to maintain a dynamic platform of data regarding maternal health issues in Brazil and has made more recent data available. According to the OOBr COVID-19 registers, Brazilian lethality among pregnant and postpartum women with ARDS due to confirmed Covid-19 is of 8.8%, with higher numbers in the state, and in the city of Rio de Janeiro: 18·3% and 20·6%, respectively. Analyzing only patients that were admitted to an ICU, lethality in the country is 26·5%, 32·7% in the state of RJ and of 36·5% in the city [22].

In our cohort, lethality, although lower than the national and state ones, can still be considered elevated. Increasing level of oxygen support, invasive and non-invasive mechanical ventilation and prone maneuver were factors associated with death. These findings suggest that severe disease and more pronounced respiratory compromise are associated with a higher risk of unfavorable outcome and that the level of oxygen support and the need for more aggressive ventilatory strategies seem to be markers of bad prognostic. Other variables associated with mortality in our study were pregnancy interruption, hemodialysis, and healthcare-related infections.

Previous studies with Brazilian data have pointed to age above 35 years old, Black race, obesity, or diabetes, and living in a peri-urban area or at more than 100 km of the notification hospital as associated factors to unfavorable outcomes, defined as admission in the ICU, need of IMV or death [19]. In this study, however, there was no statistically significant difference regarding age, race, number of comorbidities or gestational age at admission between the groups of women that died and the ones that were discharged. Interestingly, women that survived tended to live further from the ICU, although it was also not statistically significantly different. Previous studies have used composite outcomes, including ICU admission and the need of IMV, to access risk factors. Since our study comprises only patients admitted to the ICU, these differences may indicate that some risk factors may contribute to the development of more severe cases, but, among the ones in this group, they do not represent a higher risk of death.

In resonance with several previous publications [7–10], our study found a high incidence of obstetric complications, with preterm birth being the main one. The number of stillbirths/fetal losses can be considered elevated (8/101 = 7·9%). When only the events that happened during hospitalization in the ICU are considered, the numbers are similar (6/85 = 7·1%), but the outcomes of the live newborns after they were admitted to the NICU were not accessed in this study. We also didn’t have access to information of most of the newborns delivered before admission in our unit.

U.S. data collected from medical records of delivery hospitalizations during the years of 2020 and 2021 found a higher prevalence of stillbirth in women with Covid-19 in comparison with women without Covid-19 at delivery, although the numbers were smaller than ours [23]. The difference is probably due to important differences between the populations of the studies, such as disease severity and prevalence of other risk factors. Despite this, our results are in line with other reports in the literature that suggest that maternal Covid-19 is associated with a bigger risk of stillbirth [23, 24]. Considering the high frequency of *in-utero* exposure to hypoxemia, maternal stress, and several drugs used in the ICU (such as sedatives, neuromuscular blockers agents, vasoactive drugs, and antibiotics), possible consequences on the neurodevelopment of children born in the context of maternal severe Covid-19 are also a concern and should be addressed in future studies.

One important aspect that should be noted is the extremely low frequency of vaccination on our patients. Brazil’s Covid-19 vaccination strategies started in February 2021, with an age and risk-based schedule. However, pregnant, and postpartum women were only considered a priority group in April 2021. Besides that, the first reports of thrombosis with thrombocytopenia syndrome associated with some vaccine platforms halted vaccination in this population from May 2021 until July 2021, and contributed to increasing vaccine hesitation. Altogether, this scenario led to an important delay in immunization in this group, leaving already vulnerable individuals at risk of infection and susceptible to more severe disease. Recent studies have been confirming the safety of vaccination during pregnancy, without difference in perinatal outcomes compared with unvaccinated women [25–28].

Vaccination for Covid-19 in the state of RJ has reached a threshold of 70% of the target population with at least one dose in November 2021 [18]. This is the period when admissions at our unit have started to decrease, following the trends of decrease of notified cases, hospitalizations and deaths observed in the state. Even though the small number of cases does not allow making correlations, notably, none of the women admitted after this moment died or evolved to IMV.

This study has several limitations. First, although, to our knowledge, this is the biggest cohort of pregnant and postpartum women with severe Covid-19 and admission to the ICU described in the literature so far, the number of participants is small and so the number of events of interest. Therefore, some associations and differences between groups may not be found due to statistical underpower. Similarly, possible confounders may not have been detected.

Second, as it is based on data derived of medical records, it is susceptible to misclassification and there are variable amounts of missing data. To address these issues, multiple records were searched to certify the classification of the data and to decrease the number of missing data. Also, for laboratory or radiologic variables that can be affected by time or treatment, strict definitions, especially regarding the time of realization, were made. Despite that, misclassification cannot be totally ruled out.

Third, although there seems to be a time correlation between a reduction in the number of admissions and the severity of cases, and the increase in vaccine coverage in Rio de Janeiro, a causal association cannot be made. The assumption of the effect of immunization can be considered likely once several studies have demonstrated the benefit of vaccination for epidemic control [29–33]. However, transmission dynamics are frequently multifactorial, and trends in infection behavior cannot be attributed exclusively to one single factor.

Finally, as a reference unit receiving patients from multiple cities in RJ, it is expected that participants are representative of the general population and that any potential selection bias is diminished, but results may not be generalized to all pregnant women.

Our results show that pregnant and postpartum women with severe Covid-19 have high lethality and a high incidence of clinical and obstetric complications. These findings, along with other evidence in the literature that show a higher risk for severe disease, support that this population should be considered vulnerable and be prioritized on public health strategies that address Covid-19.

## Supporting information

S1 Checklist STROBE statement—Checklist of items that should be included in reports of *cohort studies*. (DOCX)
