# Supplementary material for: Severe Covid-19 in pregnant and postpartum women admitted to an intensive care unit: A retrospective cohort study
Source: PLoS One. 2023 Dec 14;18(12):e0295444. doi: 10.1371/journal.pone.0295444 (PMC10721012; doi:10.1371/journal.pone.0295444)
Supplement: S1 Checklist — (DOCX) [file pone.0295444.s001.docx]

STROBE Statement—Checklist of items that should be included in reports of ***cohort studies***

|  | Item No | Recommendation |
| --- | --- | --- |
| **Title and abstract** | 1 | 1. Indicate the study’s design with a commonly used term in the title or the abstract   **Severe Covid-19 in pregnant and postpartum women admitted to an intensive care unit: a retrospective cohort study** |
|  |  | 1. Provide in the abstract an informative and balanced summary of what was done and what was found   It’s a retrospective cohort study evaluating pregnant and postpartum women referenced to a specialized ICU between May 2020 and June 2022. Covid-19 was confirmed with RT-PCR or rapid antigen test on a nasopharyngeal swab. Variables were described by median and IQR when numerical, and by frequency and percentage when categorical. OR with 95% CI were calculated for the evaluation of factors related to death. P-values were calculated using Pearson’s ꭓ²-test, Fisher’s exact test, Wilcoxon rank sum test, and Kruskall-Wallis test, and statistical significance was established as < 0·05. Missing data were excluded. All statistical analysis were performed using R software version 4.2.2. Of the 101 admissions, 85 were of pregnant women. Obesity and systemic arterial hypertension were the most prevalent medical conditions. Sixty-six were admitted using some type of oxygen support. Forty-seven evolved to mechanical ventilation. There were 61 events considered obstetric complications, with 8 stillbirths/fetal losses. The overall lethality was 15·8%. Pregnancy interruption, NIMV, level of oxygen support at admission, prone maneuver, hemodialysis, and healthcare-related infections were factors associated with death. Evaluating the WHO 7-category ordinary scale, there was a trend of increase in the risk of death with higher punctuation, with a statistically significant difference of women with 5 (OR = 7·27; 95% IC = 1·17 – 194; p = 0·031) or 6 points (OR = 12·0; 95% IC = 1·15 – 391; p = 0·038) when compared to the ones with 3 points, i.e., of women admitted with a high-flow non-rebreathing mask or invasive mechanical ventilation, compared with the ones admitted at room air, respectively.  Page 2 |
| Introduction | | |
| Background/rationale | 2 | Explain the scientific background and rationale for the investigation being reported  Pages 3 and 4 |
| Objectives | 3 | State specific objectives, including any prespecified hypotheses  The primary objective of this study is to describe clinical, laboratory, and sociodemographic characteristics of pregnant and postpartum women with confirmed or suspected SARS-CoV-2 infection admitted to ICU. The secondary objective is to evaluate possible prognostic factors associated with death due to Covid-19 in this population.  Page 4 |
| Methods | | |
| Study design | 4 | Present key elements of study design early in the paper  It’s a retrospective exploratory cohort study evaluating pregnant and postpartum women admitted to the ICU of IEISS due to confirmed or suspected SARS-CoV-2 infection between May 2020 and June 2022.  Page 4 |
| Setting | 5 | Describe the setting, locations, and relevant dates, including periods of recruitment, exposure, follow-up, and data collection  It’s a retrospective exploratory cohort study evaluating pregnant and postpartum women admitted to the ICU of IEISS due to confirmed or suspected SARS-CoV-2 infection between May 2020 and June 2022.  Page 4 |
| Participants | 6 | 1. Give the eligibility criteria, and the sources and methods of selection of participants. Describe methods of follow-up   Consecutive admissions in the ICU were evaluated. Follow-up time was from admission to discharge of the ICU or death.  Page 5 |
|  |  | 1. For matched studies, give matching criteria and number of exposed and unexposed   NA |
| Variables | 7 | Clearly define all outcomes, exposures, predictors, potential confounders, and effect modifiers. Give diagnostic criteria, if applicable  A case was determined as confirmed if the patient presented a positive diagnostic test, either RT-PCR or rapid antigen test, on nasopharyngeal swabs. Since many women were admitted late in the disease, cases with negative results had their notification and medical records reviewed for evaluation of previous positive results and the clinical possibility of diagnosis. Cases were classified as suspected if there were clinical, epidemiological, and radiological characteristics compatible with Covid-19. Cases that had no history of a positive test and that had other etiology as more probable were excluded.  Variables of interest are described in pages 5 – 6 and below. |
| Data sources/ measurement | 8* | For each variable of interest, give sources of data and details of methods of assessment (measurement). Describe comparability of assessment methods if there is more than one group  The collected data were composed of clinical, laboratory, radiological and sociodemographic information available from the medical and multidisciplinary records of each patient.  Race registers were self-reported or defined by the attending physician. Distances were estimated in kilometres using the freely available Google Maps® app. The patients were considered immunized if, at admission, they had all recommended doses according to the type of vaccine. Partially or non-vaccinated women were considered as non-immunized. For confirmation of vaccination status, records of the National Immunization Program (Programa Nacional de Imunização - PNI), a national electronic based-system with records of all Covid-19 vaccinations of the country, were consulted.  Occurrence of ketoacidosis was defined as the concomitant presence of metabolic acidosis (serum bicarbonate < 22 mEq/L) and an elevated anion gap (> 10 mEq/L), with or without ketonuria on a urine test collected within 72h from admission. Premature births were defined as births with less than 37 weeks of pregnancy, irrespective of the mode of delivery. Stillbirths and fetal losses were counted together.  The periods of predominance of SARS-CoV-2 variants were defined according to the genomic surveillance data from the state of Rio de Janeiro. One lineage was classified as predominant when its detection rate was bigger than 90% on individuals in a period. According to those data, the original lineage was circulating in Rio de Janeiro between March and December 2020, P2 was circulating between December 2020 and February 2021, Gamma was circulating between March and June 2021, Delta was the predominant variant between August and November 2021, and Omicron was the main variant between January and March 2022 [14]. Information about the proportion of the population of the city of Rio de Janeiro vaccinated was obtained through the official epidemiological reports [15].  Pages 5 - 6 |
| Bias | 9 | Describe any efforts to address potential sources of bias  Second, as it is based on data derived of medical records, it is susceptible to misclassification and there are variable amounts of missing data. To address these issues, multiple records were searched to certify the classification of the data and to decrease the number of missing data. Also, for laboratory or radiologic variables that can be affected by time or treatment, strict definitions, especially regarding the time of realization, were made.  Pages 18 - 19 |
| Study size | 10 | Explain how the study size was arrived at  NA |
| Quantitative variables | 11 | Explain how quantitative variables were handled in the analyses. If applicable, describe which groupings were chosen and why  For descriptive analysis, numerical variables were described by median and IQR, while categorical variables were described by frequency and percentage.  Page 7 |
| Statistical methods | 12 | 1. Describe all statistical methods, including those used to control for confounding   For descriptive analysis, numerical variables were described by median and IQR, while categorical variables were described by frequency and percentage. Odds ratios (OR) with 95% confidence intervals (IC 95%) were calculated for the evaluation of factors related to death. P-values were calculated using Pearson’s ꭓ²-test, Fisher’s exact test, Wilcoxon rank sum test, and Kruskall-Wallis test, and statistical significance was established as < 0·05.  Page 7 |
|  |  | 1. Describe any methods used to examine subgroups and interactions   NA |
|  |  | 1. Explain how missing data were addressed   Missing data were excluded from the analysis.  Page 7 |
|  |  | 1. If applicable, explain how loss to follow-up was addressed   NA |
|  |  | 1. Describe any sensitivity analyses   NA |
| Results | | |
| Participants | 13* | 1. Report numbers of individuals at each stage of study—eg numbers potentially eligible, examined for eligibility, confirmed eligible, included in the study, completing follow-up, and analysed   Overall, there were 104 admissions during the established timeframe, corresponding to data from 103 different women. After reviewing the notification sheets and medical records, 3 women were excluded from the analysis since they were considered as having another cause for admission.  Page 7 |
|  |  | 1. Give reasons for non-participation at each stage   3 women were excluded from the analysis since they were considered as having another cause for admission.  Page 7 |
|  |  | 1. Consider use of a flow diagram   NA |
| Descriptive data | 14* | 1. Give characteristics of study participants (eg demographic, clinical, social) and information on exposures and potential confounders   Page 8 – Table 1 |
|  |  | 1. Indicate number of participants with missing data for each variable of interest   Included in the tables. |
|  |  | 1. Summarise follow-up time (eg, average and total amount)   The median time of hospitalization was 8 days (IQR = 4 – 21).  Page 10 |
| Outcome data | 15* | Report numbers of outcome events or summary measures over time  Overall, there were 16 deaths, 5 in the group of post-partum women and 11 in the group that was admitted pregnant, which corresponds to an overall lethality of 15·8%.  Page 12 |
| Main results | 16 | 1. Give unadjusted estimates and, if applicable, confounder-adjusted estimates and their precision (eg, 95% confidence interval). Make clear which confounders were adjusted for and why they were included   Overall, there were 16 deaths, 5 in the group of post-partum women and 11 in the group that was admitted pregnant, which corresponds to an overall lethality of 15·8%.  At the first days of hospitalization, 72 patients had metabolic acidosis, with 61 presenting an elevated anion gap, and 14 presenting both characteristics and ketonuria. During the period of the study, 6 thrombotic events were identified or suspected, and 13 patients were placed on hemodialysis. Twenty-nine patients had at least one event of healthcare-related infection.  Forty-seven women evolved to mechanical ventilation (35 pregnant and 12 post-partum), with a median time from admission to IMV of 2 days (IQR = 1 - 3) and a median time of 12 days (IQR = 6 – 23) on this modality of respiratory support. Of these 47 patients, 11 were submitted to tracheostomy and 20 were positioned in prone decubitus at least once.  Of the 85 pregnancies, 33 interruptions were identified during hospitalization. Of the women that were admitted at the post-partum period, 15 had their pregnancies interrupted in the primary health unit. In both groups, cesarian was the main mode of delivery. Of these 48 women with a history of interruption, 38 were intubated at some point during hospitalization (26 were admitted while pregnant and 12 in puerperium).  There were 61 events considered obstetric complications, mainly premature birth, with hypertensive disorders of pregnancy being the second most frequent. There were 8 stillbirths/fetal losses.  All women that died needed mechanical ventilation. Pregnancy interruption (OR = 9·70; 95% IC = 2·46 – 70·4; p < 0·001), receiving NIMV (OR = 17·1; 95% IC = 3·20 – 427; p < 0·001), being submitted to prone maneuver (OR = 3·82; 95% IC = 1·04 – 15·7; p = 0·043), receiving hemodialysis (OR = 6·33; 95% IC = 1·69 – 23·7; p = 0·007), and at least one episode of healthcare-related infection (OR = 6·69; 95% IC = 2·08 – 24·4; p = 0·001) were factors statistically associated with death (Table 4).  Evaluating the WHO 7-category ordinary scale, there was a trend of increase in the risk of death with higher punctuation, with a statistically significant difference of women with 5 (OR = 7·27; 95% IC = 1·17 – 194; p = 0·031) or 6 points (OR = 12·0; 95% IC = 1·15 – 391; p = 0·038) when compared to the ones with 3 points, i.e., of women admitted with a high-flow non-rebreathing mask or invasive mechanical ventilation, compared with the ones admitted at room air, respectively.  When analyzing the distribution of fatal cases according to variants' circulation, the period with the highest lethality was the one when Delta was the predominant variant, with 5 deaths among the 21 admissions in the period (23·8%), followed by the period when the Gamma variant was the main one (5/48; 10·4%). There were no deaths during the P2 and Omicron waves. There were no deaths registered from October 2021 onwards.  Pages 11 - 15 |
|  |  | 1. Report category boundaries when continuous variables were categorized   NA |
|  |  | 1. If relevant, consider translating estimates of relative risk into absolute risk for a meaningful time period   NA |
| Other analyses | 17 | Report other analyses done—eg analyses of subgroups and interactions, and sensitivity analyses  NA |
| Discussion | | |
| Key results | 18 | Summarise key results with reference to study objectives  Our results show that severe Covid-19 in women that are pregnant or in the postpartum period is associated with high lethality.  Increasing level of oxygen support, invasive and non-invasive mechanical ventilation and prone maneuver were factors associated with death. These findings suggest that severe disease and more accentuated respiratory compromise are associated with a higher risk of unfavorable outcome and that the level of oxygen support and the need for more aggressive ventilatory strategies seem to be markers of bad prognostic. Other variables associated with mortality in our study were pregnancy interruption, hemodialysis, and healthcare-related infections.  Pages 15 |
| Limitations | 19 | Discuss limitations of the study, taking into account sources of potential bias or imprecision. Discuss both direction and magnitude of any potential bias  This study has several limitations. First, although, to our knowledge, this is the biggest cohort of pregnant and postpartum women with severe COVID and admission to the ICU described in the literature so far, the number of participants is small and so the number of events of interest. Therefore, some associations and differences between groups may not be found due to statistical underpower. Similarly, possible confounders may not have been detected.  Second, as it is based on data derived of medical records, it is susceptible to misclassification and there are variable amounts of missing data. To address these issues, multiple records were searched to certify the classification of the data and to decrease the number of missing data. Also, for laboratory or radiologic variables that can be affected by time or treatment, strict definitions, especially regarding the time of realization, were made. Despite that, misclassification cannot be totally ruled out.  Third, although there seems to be a time correlation between a reduction in the number of admissions and the severity of cases, and the increase in vaccine coverage in Rio de Janeiro, a causal association cannot be made. The assumption of the effect of immunization can be considered likely once several studies have demonstrated the benefit of vaccination for epidemic control [26,27,28,29,30]. However, transmission dynamics are frequently multifactorial, and trends in infection behavior cannot be attributed exclusively to one single factor.  Finally, as a reference unit receiving patients from multiple cities in RJ, it is expected that participants are representative of the general population and that any potential selection bias is diminished, but results may not be generalized to all pregnant women.  Pages 18 - 19 |
| Interpretation | 20 | Give a cautious overall interpretation of results considering objectives, limitations, multiplicity of analyses, results from similar studies, and other relevant evidence  Pages 15 - 19 |
| Generalisability | 21 | Discuss the generalisability (external validity) of the study results  Finally, as a reference unit receiving patients from multiple cities in RJ, it is expected that participants are representative of the general population and that any potential selection bias is diminished, but results may not be generalized to all pregnant women.  Page 19 |
| Other information | | |
| Funding | 22 | Give the source of funding and the role of the funders for the present study and, if applicable, for the original study on which the present article is based  There was no funding for this study. Not included in this version of the manuscript, according to Journal instructions for publication. |

*Give information separately for exposed and unexposed groups.
